# Heavy metal content of over-the-counter toothpastes—a systematic review of *in vitro* studies

**DOI:** 10.3389/fdmed.2025.1543972

**Published:** 2025-03-26

**Authors:** Kavery Chengappa, Ashwini Rao, Ramya Shenoy, Mithun Pai, Praveen Jodalli, Avinash BR

**Affiliations:** Manipal College of Dental Sciences Mangalore, Manipal Academy of Higher Education, Manipal, Karnataka, India

**Keywords:** bioaccumulation, environmental sustainability, heavy metals, systematic review, toothpaste

## Abstract

**Background:**

Heavy metal contamination of the environment has become a global problem because of their toxicity, environmental persistence, bioaccumulation, and biomagnification. The daily use of toothpastes containing heavy metals can be a threat to the environment since they can bioaccumulate and reach toxic proportions, affecting water, soil and living organisms.

**Methods:**

This systematic review was performed to identify, assess, and compile information from the literature that looks into the possibility of heavy metals in toothpastes. The keyword search resulted in a total of 9,409 articles. After removal of duplicates, screening of titles and the abstracts, eleven studies that met the inclusion criteria were included. The quality of the studies was assessed using the Quality Assessment Tool for *in vitro* Studies (QUIN).

**Results:**

All eleven included studies reported the presence of heavy metals in one form or the other. This systematic review provides evidence for the presence of heavy metals in toothpastes from around the world.

**Conclusions:**

In addition to being harmful to the consumer's health, these toothpastes are also harmful to the environment. Toothpaste containing heavy metals contributes to the already high level of heavy metal pollution in the environment from industrial and agricultural processes. There is a need for specific guidelines on the limits for heavy metals in toothpastes, with a clear distinction between essential and nonessential metals. More evidence from all parts of the world is needed to understand the gravity of the situation and to carry out remedial measures.

**Systematic Review Registration:**

https://doi.org/10.17605/OSF.IO/V9P2D

## 1 Introduction

Heavy metals are among the many pollutants that have been of concern in recent years. They are elements that are naturally present in the Earth's crust in small quantities and are often understood to be those with a density greater than 5 g/c.c (1). Their concentration in the environment has increased due to human activities such as industrial processes, agricultural activities, construction and dredging, domestic activities and the discharge of untreated waste (2). Although nonessential heavy metals have no known biological function and can be highly hazardous if allowed to accumulate, essential heavy metals, despite their known biological roles, can still be dangerous above threshold amounts. Since heavy metals are persistent, harmful to the environment, nonbiodegradable and bioaccumulate, they pose a significant threat to ecology (3).

A distinct contributor that has emerged recently is that of personal care and cosmetic products (4). Cosmetics of innumerable categories, such as hair shampoos, facial creams, gels, soaps, toothpastes and many others, have been shown to be the prime source of unregulated heavy metals released into the environment (5). The high concentration of these heavy metals in tainted personal care products has become a serious problem and one such personal care product that is irreplaceable for the daily life of millions of people worldwide is toothpaste. Studies (6–8) have shown that toothpastes that are commonly used daily as an inevitable component of regular oral hygiene practice have high concentrations of heavy metals. If toothpastes contain heavy metals even in small concentrations, they could bioaccumulate and reach toxic proportions over a long period of time.

Heavy metals at toxic concentrations can lead to the formation of free radicals, altering homeostasis, and lipid peroxidation, subsequently causing modulation of the cell cycle, apoptosis or carcinogenesis (9). Arsenic has perilous effects on the human body, causing alterations at the cellular level and it is even known to induce skin cancer (10). Cadmium has shown multitissue carcinogenic potential by affecting the lungs, kidneys and pancreas upon chronic exposure (11). Irritability, appetite loss, blue‒black gum deposits, weight loss, vomiting, constipation, stomach pain, anaemia, and renal failure are the signs and symptoms of lead poisoning and mercury causes oxidative stress and cellular damage (1).

**Figure 1:**
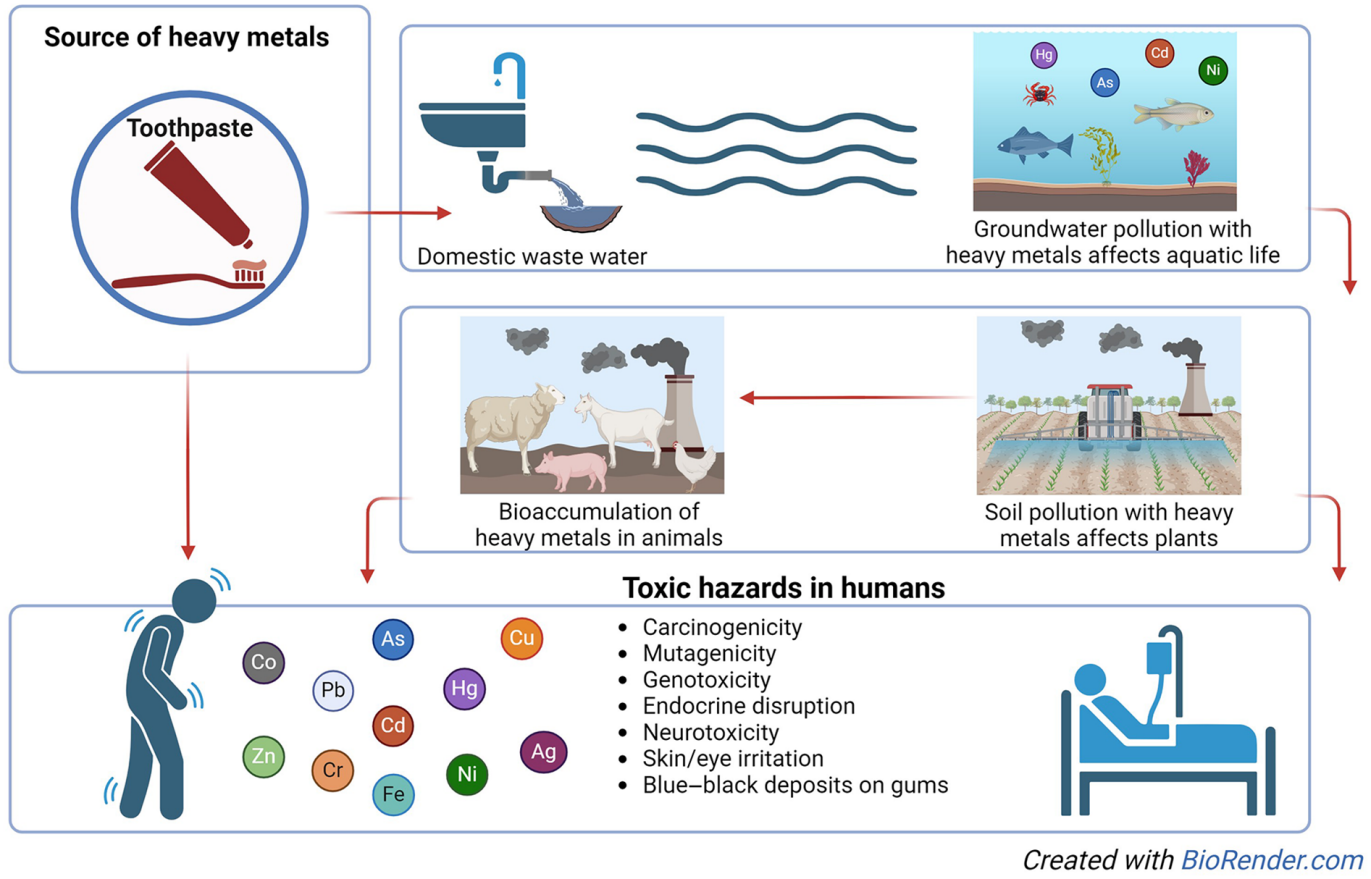
Life course of heavy metals in toothpastes.

The toxicity of these elements, together with their environmental persistence, bioaccumulation, and biomagnification in food chains, makes heavy metal contamination of the environment a global problem (12). The daily use of toothpastes containing heavy metals can be a threat to the environment since they can bioaccumulate and reach toxic proportions (Figure 1).

## 2 Rationale

Although heavy metals are naturally present on earth, these substances are also continuously added by human activities to the environment, contaminating the air, water, and soil constituting a serious threat to the health of humans. Studies have shown the presence of heavy metals in personal care products and toothpastes (6, 7). Toothpastes, especially their inevitable daily usage, could be major contributors to heavy metal contamination. Although only a pea-sized quantity is used per person per brushing, their daily use across the population could result not only in bioaccumulation in the body with hazardous effects (13) but also result in contamination of the environment.

## 3 Objective

To identify, assess, and compile information in a methodical manner from the literature that looks into the possibility of heavy metals in over-the-counter toothpastes.

## 4 Methodology

Since this was a systematic review, the Declaration of Helsinki is not applicable. However, the Institutional research committee approval has been obtained (No: 685/2024) and the protocol has been registered at open science framework (OSF) registries (14).

### 4.1 Eligibility criteria

#### 4.1.1 Inclusion criteria

The PICOS criteria were used for including studies for the review:

i. (P) Population/participants and conditions of interest: Over-the-counter toothpaste samples
ii. (I) Interventions/exposures: None
iii. (C) Comparisons or control groups: Any or no comparison
iv. (O) Outcomes of interest: Presence and types of heavy metals
v. (S) Study designs: *in vitro* studies

#### 4.1.2 Exclusion criteria

Studies that were published in languages other than English, those that lacked full texts or abstracts and studies not fitting the PICO framework, such as randomized clinical trials, were excluded.

### 4.2 Information sources

The electronic databases of Google Scholar, Embase, Springer Link, PubMed, Web of Science, and Scopus were searched. There was also a manual search of the papers using references.

### 4.3 Search strategy

The search strategy involved the use of the following key terms involving the Boolean “AND” operator: “heavy metals” AND toothpastes OR “heavy metals” AND dentifrices, sort by relevance, Filters: English. All articles obtained through the keyword search were selected for the data collection process (Supplementary Table 1).

### 4.4 Data collection process

Two reviewers, reviewer number 1 (KC) and reviewer number 2 (AR), collected data for this review. The articles were separately examined by both reviewers. A third reviewer (RS) assisted in resolving any disagreements that may have existed. Covidence® software was used to extract the data.

### 4.5 Data items

The following information was obtained: the presence, type and concentration of heavy metals in the toothpastes. The data extraction template by Covidence was customized for this review, and information about the methodology, instrument used, and type and concentration of heavy metals identified was carefully extracted from each included study. The standards of the Preferred Reporting Items for Systematic Reviews and Meta-Analyses (PRISMA) 2020 (15) were used to report the findings.

### 4.6 Quality assessment

The Quality Assessment Tool for *in vitro* Studies (QUIN) was utilized to evaluate the quality of the chosen studies (16). The QUIN tool has twelve criteria that need to be assessed and then an allotted point value with 2 points given for adequately specified, one for inadequately specified and no points for not specified. Of the twelve criteria given by the QUIN tool, only nine criteria were considered for this quality assessment. The two excluded criteria, operator details and randomization, were not relevant for this study design, and another criterion, details of the comparison group, was excluded since no comparison group was needed, as specified in the PICOS criteria for this systematic review. The quality of the papers was independently assessed by the first reviewer (KC) and the second reviewer (AR). If there were any differences, discussions were made to find a solution.

## 5 Results

### 5.1 Study selection

The keyword search resulted in a total of 9,409 articles, of which 8,730 were from Google Scholar, 376 from PubMed/MEDLINE, 210 from Scopus, 69 from Springer Link, 12 from Embase and 12 from Web of Science.

**Figure 2:**
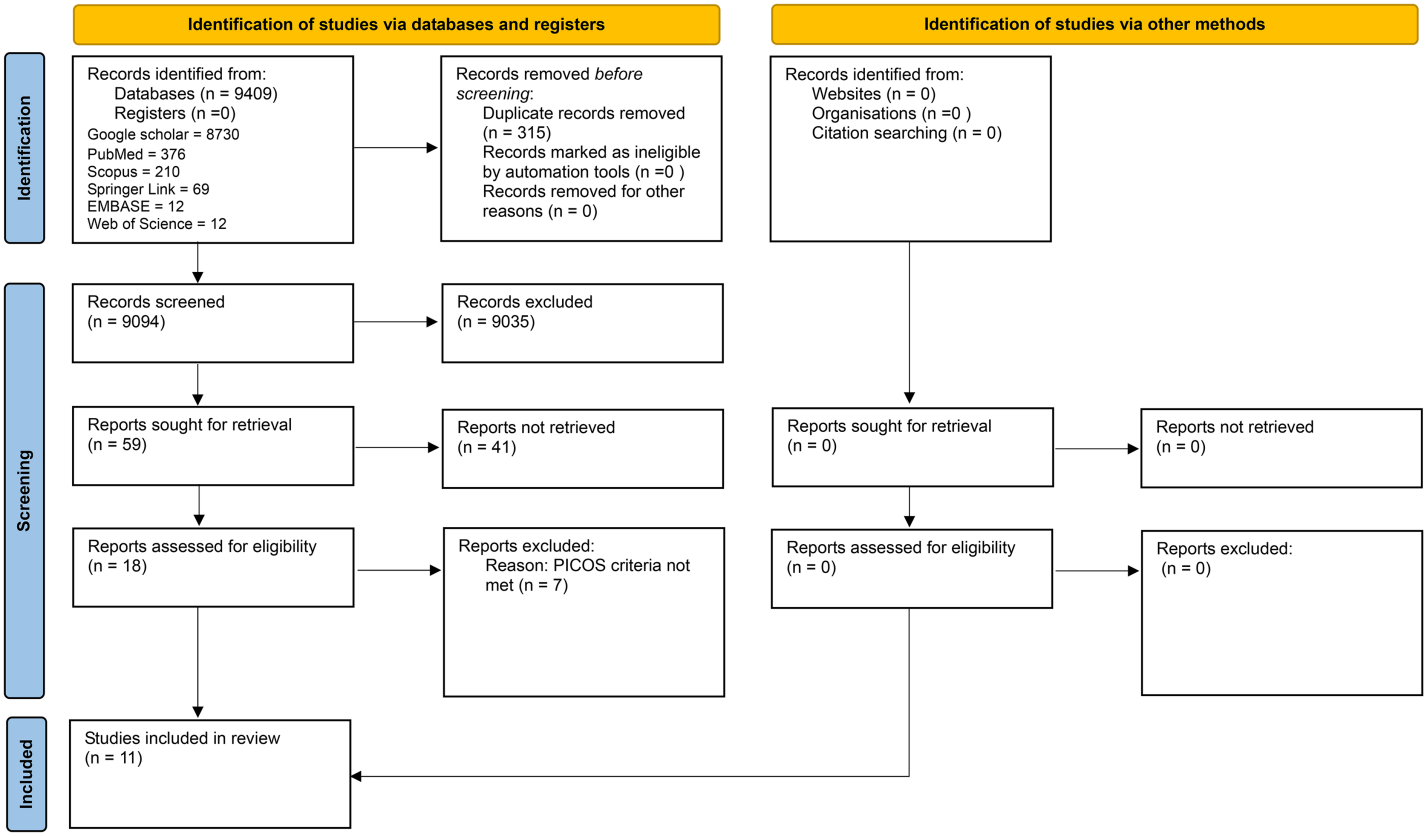
PRISMA 2020 flow diagram.

When the 315 duplicates were identified and removed, 9,094 articles remained. These articles were title screened, and 59 articles were included for the next step of abstract screening. Among the 59 articles, 18 were eligible for full-text review. On the basis of the inclusion criteria, only eleven studies that met the PICOS criteria were included in this systematic review (Figure 2). The Cohen's Kappa score, measuring the inter-rater reliability between authors, was found to be 1, indicating perfect agreement.

### 5.2 Characteristics of the included studies

Among the eleven included studies, location-wise categorization resulted in statistics of five studies originating from Nigeria (17–21), two from Saudi Arabia (6, 8), and one each from Bangladesh (22), Iraq (23), Malta (7) and Pakistan (24). Among the eleven studies included, eight utilized the atomic absorption spectrophotometer (AAS) method for the detection of heavy metals (17–24). Two studies used inductively coupled plasma‒mass spectrometry (6, 8), and one study used a microwave plasma‒atomic emission spectrometer (7) for the detection of heavy metals (Table 1).

**Table 1:** Characteristics of the included studies.

Sl No.	Author and year	Place	Sample size	Methodology used	Heavy metals detected
1.	Odukudu et al. (2013)	Nigeria	11	Flame Atomic Absorption Spectrophotometer (AAS)	Cadmium, Chromium, lead, Nickel, Copper, iron and Zinc
2.	Ideriah et al. (2016)	Nigeria	9	Atomic Absorption Spectrophotometer (AAS)	Iron, Chromium
3.	Orisakwe et al. (2016)	Nigeria	35	Flame Atomic Absorption Spectrophotometry 205A (AAS)	Lead, cobalt, chromium, nickel and cadmium
4.	Salama (2016)	Saudi Arabia	4	Inductively coupled plasma mass spectrometry (ICP–MS)	Lead, aluminium, cadmium, cobalt, chromium, copper, manganese, nickel, mercury, and arsenic
5.	Vella and Attard (2019)	Malta	9	Microwave Plasma-Atomic Emission Spectrometer (MP—AES)	Silver, chromium, copper, nickel, lead, tin, zinc, iron
6.	Arshad et al. (2020)	Pakistan	18	Atomic Absorption Spectrophotometer (AAS)	Cadmium
7.	Paul et al. (2020)	Bangladesh	10	Atomic Absorption Spectrophotometer (AAS)	Copper, lead, arsenic
8.	Ogidi and Agbo (2021)	Nigeria	5	Atomic Absorption Spectrometer Model AA-7000 (AAS)	Zinc
9.	Almukainzi et al. (2022)	Saudi Arabia	2	Inductively Coupled Plasma–Mass Spectrometry (ICP–MS)	Chromium, iron, cobalt, nickel, copper, zinc, arsenic, cadmium, lead
10.	Valentine and Ozioma (2022)	Nigeria	5	Perkin Elmer Atomic Absorption Spectrophotometer (AAS)	Iron, lead, copper, chromium, zinc, nickel, cadmium
11.	Lawi et al. (2023)	Iraq	10	Flame Atomic Absorption Spectrophotometer (AAS)	Zinc, Iron, Lead

### 5.3 Heavy metals identified

Studies have examined various heavy metals, both essential and toxic. Cobalt, zinc, chromium, copper, iron, and nickel were the essential heavy metals, and cadmium, lead, mercury, arsenic and silver were the toxic heavy metals analysed in these studies. Odukudu et al. (17), in their study on toothpastes in Nigeria, reported the presence of seven heavy metals, two of which were toxic metals, cadmium and lead, whose concentrations were 0.035 and 0.02 ppm, respectively. The remaining heavy metals identified were zinc, chromium, copper, iron and nickel.

Although Ideriah et al. (18), in their study on toothpastes in Nigeria, reported the presence of iron in all the toothpastes, the presence of chromium was found only in five toothpastes, and the concentration was found to be very low, <0.006 ppm. Orisawke et al. (19), in their study in Nigeria, conducted studies on 35 toothpastes, examined the presence of five types of heavy metals and reported high concentrations of the toxic heavy metal lead in all 35 samples and cadmium in 26 samples. The daily intake of cadmium was estimated to be high for one sample of toothpaste, which exceeded the stated upper permissible level. The essential heavy metals cobalt and nickel were found in high concentrations in all the samples, and chromium was found in 24 samples.

Salama (6) examined the presence of eight heavy metals in four toothpastes and reported the presence of the toxic heavy metals cadmium, lead and arsenic in all the toothpastes. The concentration of cadmium ranged from 2.008 ppb to 55.28 ppb, that of lead ranged from 1,856 ppb to 6,313 ppb, and that of arsenic ranged from 0.6 ppb to 26.94 ppb. Mercury was also found in three of the four toothpastes, ranging from 3.34 ppb to 13.14 ppb. The essential heavy metals cobalt, chromium, copper and nickel were also present in all four toothpaste samples tested.

Vella and Attard (7), in their study of nine toothpastes in Malta, detected toxic heavy metals, lead and silver in all the samples, with concentrations of lead ranging from 2.37 ppm to 12.04 ppm and those of silver ranging from 2 ppm to 5.12 ppm. Essential heavy metals. Chromium, iron and nickel were also detected in all nine samples, and zinc was detected in eight of the nine toothpastes studied. However, cadmium and mercury were not detected in any of the samples.

Arshad et al. (24) studied eighteen toothpastes from Pakistan for the presence of the toxic heavy metal cadmium and detected cadmium in eleven of the eighteen samples examined, with concentrations ranging from 0.0125 ppm to 1.392 ppm. Paul et al. (22) studied ten toothpastes in Bangladesh and reported the presence of the toxic heavy metals lead and arsenic, which ranged from 0.27–2.12 ppm and 0.02–0.637 ppm, respectively, in all the toothpastes. The essential heavy metal copper was also found in all the toothpastes examined. Ogidi and Agbo (20) studied five toothpaste samples in Nigeria and reported the presence of zinc in all the samples. The range was between 1.19 ppm and 3.08 ppm in three toothpaste samples, but it was reported to be very high in the other two samples, at 81.27 ppm and 84.67 ppm.

Almukainzi et al. (8), in their study on two toothpastes in Saudi Arabia, reported the presence of the toxic heavy metals cadmium, lead and arsenic in both samples at concentrations of 8.8 ppm to 9.19 ppm, 75.86–78.31 ppm and 209.33–221.96 ppm, respectively. They also reported very high concentrations of the essential heavy metals cobalt, zinc, chromium, copper, iron and nickel in both samples.

In their study of ten toothpastes in Nigeria, Valentine and Ozioma (21) reported the presence of the toxic heavy metals cadmium and lead in two of the ten toothpastes, which ranged from 0.01–0.13 ppm and 0.01–0.02 ppm, respectively. The essential heavy metals zinc and iron were found in eight toothpastes, chromium and nickel were found in six samples, and copper was found in five samples.

In their study of toothpastes in Iraq, Lawi et al. (23), reported the presence of the toxic heavy metal lead in all ten samples in the range of 1–12.05 ppm. The essential heavy metals zinc and iron were also detected in all ten samples in the ranges of 1.59 ppm to 402.34 ppm and 36.75 ppm to 654.24 ppm, respectively (Supplementary Table 2).

### 5.4 Quality of the included studies

Upon evaluating the quality of the chosen studies with the Quality Assessment Tool for *in vitro* Studies (QUIN) (16), it was found that all the studies specified the aim/objective, detailed explanation of methodology, method of measurement of outcome and presentation of results. However, most of the studies lacked a thorough explanation of the sample size calculation and sampling technique, operator details, randomization, outcome assessor details, and blinding. The final scores ranged between 33.33 and 55.55. Seven of the eleven studies had a high risk of bias, and four studies had a medium risk of bias (Table 2).

**Table 2:** Quality assessment of the included articles using the quality assessment tool for *in vitro* studies (QUIN**).**

Sl No.	Criteria	Odukudu et al. (2013)	Ideriah et al. (2016)	Orisakwe et al. (2016)	Salama (2016)	Vella and Attard (2019)	Arshad et al. (2020)	Paul et al. (2020)	Ogidi and Agbo (2021)	Almukainzi et al. (2022)	Valentine and Ozioma (2022)	Lawi et al. (2023)
1.	Clearly stated aims/objectives	1	1	1	2	2	1	1	1	1	2	2
2.	Detailed explanation of sample size calculation	1	1	0	1	1	0	0	0	0	0	0
3.	Detailed explanation of sampling technique	0	0	1	0	1	0	0	0	1	0	0
4.	Details of comparison group	Excluded										
5.	Detailed explanation of methodology	2	2	2	2	2	2	2	2	2	2	2
6.	Operator details	Excluded										
7.	Randomization	Excluded										
8.	Method of measurement of outcome	2	2	2	1	2	2	2	1	1	2	2
9.	Outcome assessor details	0	0	0	0	0	0	0	0	0	0	0
10.	Blinding	0	0	0	0	0	0	0	0	0	0	0
11.	Statistical analysis	2	0	0	0	0	0	0	0	0	2	2
12.	Presentation of results	2	2	2	1	2	2	2	2	2	2	2
	SUM	10	8	7	7	10	7	7	6	7	10	10
	FINAL SCORE	55.55	44.4	38.8	38.8	55.55	38.8	38.8	33.3	38.8	55.55	55.55
	RISK OF BIAS	Medium	High	High	High	Medium	High	High	High	High	Medium	Medium

## 6 Discussion

The characteristics and toxicity of a number of heavy metals and other chemical elements that naturally occur in the Earth's crust have been understood for thousands of years. This ubiquity of heavy metals in daily life may lead to many exposures that could harm humans. While cadmium has been reported to be added to cosmetics as a colouring agent, lead and arsenic are recognized contaminants (4). Mercury has been detected in considerable amounts in skin-lightening creams, although it has rarely been found in other cosmetics (4). Although heavy metals might make their way into these products unintentionally through contaminated raw materials, heavy metals have been added to dental care products, especially silver diamine fluoride, because of their anticaries activity (25). The application of cosmetics and other personal care items to the skin can dramatically accelerate the gradual accumulation of heavy metals to hazardous levels. Therefore, regulatory agencies prescribe limits beyond which these metals are not to be present in cosmetics and toothpastes.

The Food and Drug Administration (FDA) in the United States has set limitations on arsenic at 3 ppm, mercury at 1 ppm, lead at 20 ppm and chromium at 50 ppm (26). Limits were established by the World Health Organization (WHO) for lead at 10 ppm, cadmium at 0.3 ppm and mercury at 1 ppm (27). The EU has set limits of 0.5 for lead and mercury, 0.1 for arsenic and 1.0 ppm for chromium (7). The Canadian government has set limits of 20 ppm for lead, 5 ppm for arsenic and cadmium and 1 ppm for mercury for cosmetic products, and the maximum acceptable concentrations for toothpastes are 1 ppm for lead, 0.5 ppm for arsenic, 0.1 ppm for cadmium and 0.2 ppm for mercury (28). In India, the Bureau of Indian Standards (BIS) specified that the maximum limit of heavy metals in toothpaste should not exceed 20 ppm (29).

All eleven studies included in this systematic review reported the presence of heavy metals in one form or the other. Odukudu et al. (17), have expressed concern about the levels of cadmium and chromium in the toothpastes since these elements were ideally not supposed to be present in these products. Orisakwe et al. (19), reported that despite the fact that the upper limit, the target hazard quotient and daily consumption rates were all within normal ranges, the high lead levels in a few of the toothpastes raise serious public health concerns. Salama (6) reported the presence of cadmium, lead, mercury and arsenic in all toothpastes in Saudi Arabia and has predicted that if these products are used consistently, the amount of heavy metals in the human body may rise above tolerable levels.

Vella and Attard (7) reported that the concentrations of lead and nickel were above the upper limit set by US and EU regulations. Arshad et al. (24), reported that eleven samples contained cadmium at concentrations ranging from 0.0037 to as high as 1.39 ppm. Paul et al. (22), reported that the average concentrations of arsenic, copper, and lead were within the permissible range for toothpaste, but the average concentration of arsenic reportedly exceeded the threshold value for drinking water. According to Ogidi and Agbo (20), three of the samples presented levels that were considerably short of the daily maximum for zinc intake, whereas the remaining two samples presented substantially higher values than the maximum amount of other heavy metals allowed in toothpastes.

Almukainzi et al. (8), reported the presence of the toxic heavy metals cadmium, lead and arsenic in samples. They also reported very high concentrations of zinc, chromium, copper, iron, nickel and cobalt. Valentine and Ozioma (21) reported the presence of cadmium, lead, zinc, iron, chromium, nickel and copper in samples. Lawi et al. (23), reported the presence of lead, zinc and iron in all the samples studied and concluded that the levels of zinc were higher than the WHO guidelines in three out of the ten toothpastes studied. Very high concentrations of zinc were found in one sample, and high concentrations of iron were found in all the samples.

### 6.1 Limitations

This systematic review was limited to studies published in the English language, because of the difficulty in article translation and interpretation by the reviewers. This could have led to language bias, affecting the findings of this review. Evidence for the presence of heavy metals in toothpastes was available only from Nigeria, Malta, Bangladesh, Pakistan and Saudi Arabia, and among these studies, seven had a high risk of bias, and four had a medium risk of bias. Since this review is based solely on *in vitro* studies, this limits the ability to assess the real effects of these toothpastes on the human body. Additionally, the absence of specific brand names of toothpastes in some studies could affect the applicability of the results.

The methodology of the review was robust, with the use of PICOS criteria for inclusion of studies, the customized Covidence data extraction template for data extraction and the QUIN (16) for assessing the quality of the selected studies. The findings were reported as per the Preferred Reporting Items for Systematic Reviews and Meta-Analyses (PRISMA) 2020 (15) (Supplementary Table 3).

Even though some of these elements are vital to human health, when their quantities rise above the safety threshold, they become toxic. Permissible limits are given for safeguarding the health of an individual. However, their mere presence even in very small quantities can be detrimental to the environment since they can bioaccumulate when they are disposed into the environment repeatedly. Since heavy metals have low mobility and limited degradability, once introduced into the environment, they can persist for long periods and accumulate over time. Therefore, even small quantities, such as those present in toothpastes, can add to its bioaccumulation in humans and in the environment.

## 7 Conclusions

This systematic review aimed to obtain evidence for the presence of heavy metal contaminants in toothpastes by reviewing studies conducted around the world. The methodological quality of the included studies were poor with seven out of eleven studies showing a high risk of bias, which could weaken the robustness of the findings. However, this review has demonstrated the presence of heavy metals at varying concentrations in many toothpastes. Although some heavy metals such as cadmium, lead, mercury, arsenic and silver are toxic and must not be present in toothpastes, there are other heavy metals that are essential in small quantities but could result in adverse effects if present in excess. The FDA (26) has set limitations on arsenic at 3 ppm, mercury at 1 ppm, lead at 20 ppm and chromium at 50 ppm whereas the WHO (27) limits the presence of lead at 10 ppm, cadmium at 0.3 ppm and mercury at 1 ppm in toothpastes. In the present review, some of the toothpastes in the study by Almukainzi et al. (8) reported the presence of arsenic beyond the permissible FDA levels and some (19) reported the presence of lead beyond permissible levels given by FDA and the WHO, and both of these are toxic heavy metals. Another toxic heavy metal cadmium was also reported to be present in 12 out of 35 toothpastes tested by Orisakwe et al. (19), clearly in excess of the WHO guidelines of 0.3 ppm. Owing to the large-scale use and reach of toothpastes, the presence of heavy metals could pose a potential threat to the health of the consumer. They could also add to the existing burden of heavy metals being released from industrial and agricultural processes, thus jeopardizing the stability of the environment over time. It is imperative to recognize and ensure action so that toothpastes, which aid in promoting health and preventing illness, do not contribute to it.

## 8 Implications for practice, policy, and future research

• This review shows that both healthcare professionals and consumers need to be vigilant and cautious regarding the selection and use of safer toothpaste products.
• There is a need for specific guidelines on the limits for heavy metals in toothpastes, with a clear distinction between essential and nonessential metals.
• Toothpastes should not exceed the acceptable safety limits prescribed by regulatory agencies. Stringent quality assurance processes need to be developed and put into place by manufacturers and strictly assessed and evaluated by regulators.
• More studies from different parts of the world are needed to identify the presence of heavy metals in toothpastes.
