# Supplementary material for: Heavy metal content of over-the-counter toothpastes—a systematic review of in vitro studies
Source: Front Dent Med. 2025 Mar 26;6:1543972. doi: 10.3389/fdmed.2025.1543972 (PMC11979237; doi:10.3389/fdmed.2025.1543972)
Supplement: Supplementary file 1 [file Table1.doc]

**Supplementary Table 1. Search strategy**

|  | **Databases** | **Search strategy** | **Records identified** |
| --- | --- | --- | --- |
|  | Google scholar | heavy metals AND toothpastes OR heavy metals AND dentifrices | **8730** |
|  | PubMed | heavy metals AND toothpastes OR heavy metals AND dentifrices | **376** |
|  | Scopus | TITLE-ABS-KEY ( "heavy metals" AND toothpastes ) OR "heavy metals" AND dentifrices | **210** |
|  | Springer Link | “heavy metals” AND toothpastes OR “heavy metals” AND dentifrices | **69** |
|  | EMBASE | (('heavy metals'/exp OR 'heavy metals') AND ('toothpastes'/exp OR toothpastes) OR 'heavy metals'/exp OR 'heavy metals') AND ('dentifrices'/exp OR dentifrices) | **12** |
|  | Web of Science | "heavy metals" AND toothpastes  OR "heavy metals" AND dentifrices (All Fields) | **12** |
|  |  | **TOTAL** | **9409** |
