# Supplementary material for: Heavy metal content of over-the-counter toothpastes—a systematic review of in vitro studies
Source: Front Dent Med. 2025 Mar 26;6:1543972. doi: 10.3389/fdmed.2025.1543972 (PMC11979237; doi:10.3389/fdmed.2025.1543972)
Supplement: Supplementary file 2 [file Table2.doc]

**Supplementary Table 2. Summary of findings of the included studies**

| Author | Year | Place | Toothpaste | **Toxic heavy metals** | | | | | **Essential heavy metals** | | | | | |
| --- | --- | --- | --- | --- | --- | --- | --- | --- | --- | --- | --- | --- | --- | --- |
|  |  |  |  | Cadmium | Lead | Mercury | Arsenic | Silver | Cobalt | Zinc | Chromium | Copper | Iron | Nickel |
| Odukudu et al | 2013 | Nigeria | TP 1 | 0.035±0.23 | 0.02±0.016 | Not tested | Not tested | Not tested | Not tested | 0.255±0.108 | 0.015±0.009 | 0.23±0.08 | 0.608±0.068 | 0.035±0.015 |
| Ideriah et al | 2016 | Nigeria | TP 1 | Not tested | Not tested | Not tested | Not tested | Not tested | Not tested | Not tested | <0.006 | Not tested | 0.32 | Not tested |
| Ideriah et al | 2016 | Nigeria | TP 2 | Not tested | Not tested | Not tested | Not tested | Not tested | Not tested | Not tested | <0.006 | Not tested | 0.23 | Not tested |
| Ideriah et al | 2016 | Nigeria | TP 3 | Not tested | Not tested | Not tested | Not tested | Not tested | Not tested | Not tested | <0.006 | Not tested | <0.03 | Not tested |
| Ideriah et al | 2016 | Nigeria | TP 4 | Not tested | Not tested | Not tested | Not tested | Not tested | Not tested | Not tested | <0.006 | Not tested | 0.45 | Not tested |
| Ideriah et al | 2016 | Nigeria | TP 5 | Not tested | Not tested | Not tested | Not tested | Not tested | Not tested | Not tested | <0.006 | Not tested | 0.42 | Not tested |
| Ideriah et al | 2016 | Nigeria | TP 6 | Not tested | Not tested | Not tested | Not tested | Not tested | Not tested | Not tested | 0 | Not tested | 1.62 | Not tested |
| Ideriah et al | 2016 | Nigeria | TP 7 | Not tested | Not tested | Not tested | Not tested | Not tested | Not tested | Not tested | 0 | Not tested | 0.12 | Not tested |
| Ideriah et al | 2016 | Nigeria | TP 8 | Not tested | Not tested | Not tested | Not tested | Not tested | Not tested | Not tested | 0 | Not tested | 0.23 | Not tested |
| Ideriah et al | 2016 | Nigeria | TP 9 | Not tested | Not tested | Not tested | Not tested | Not tested | Not tested | Not tested | 0 | Not tested | 0.25 | Not tested |
| Orisakwe et al | 2016 | Nigeria | Close Up Complete 8 Actions | 0.043 | 11.033 | Not tested | Not tested | Not tested | 1.989 | Not tested | 0 | Not tested | Not tested | 14.505 |
| Orisakwe et al | 2016 | Nigeria | Close Up Fine Free Dual Sensation | 0 | 11.412 | Not tested | Not tested | Not tested | 2.869 | Not tested | 0.001 | Not tested | Not tested | 14.312 |
| Orisakwe et al | 2016 | Nigeria | Close Up Herbal (Family Teeth) | 0.043 | 14.871 | Not tested | Not tested | Not tested | 5.992 | Not tested | 3.897 | Not tested | Not tested | 17.05 |
| Orisakwe et al | 2016 | Nigeria | Close Up Loves Deep | 0 | 12.116 | Not tested | Not tested | Not tested | 4.769 | Not tested | 0 | Not tested | Not tested | 12.101 |
| Orisakwe et al | 2016 | Nigeria | Close Up Red Hot | 0 | 13.103 | Not tested | Not tested | Not tested | 3.428 | Not tested | 0.002 | Not tested | Not tested | 13.789 |
| Orisakwe et al | 2016 | Nigeria | Close Up White On | 0.001 | 10.689 | Not tested | Not tested | Not tested | 5.137 | Not tested | 0 | Not tested | Not tested | 15.121 |
| Orisakwe et al | 2016 | Nigeria | Dabour Herbal (Mint x Lemon) | 0.329 | 17.888 | Not tested | Not tested | Not tested | 8.178 | Not tested | 0 | Not tested | Not tested | 8.573 |
| Orisakwe et al | 2016 | Nigeria | Macleans Complete Care | 0 | 13.004 | Not tested | Not tested | Not tested | 5.108 | Not tested | 1.024 | Not tested | Not tested | 6.798 |
| Orisakwe et al | 2016 | Nigeria | Macleans Herbal | 0 | 8.768 | Not tested | Not tested | Not tested | 5.032 | Not tested | 2.211 | Not tested | Not tested | 7.024 |
| Orisakwe et al | 2016 | Nigeria | Medicamente Dabour Natural | 0 | 8.699 | Not tested | Not tested | Not tested | 5.328 | Not tested | 0.542 | Not tested | Not tested | 9.302 |
| Orisakwe et al | 2016 | Nigeria | Milk Teeth Macleans | 0.043 | 11.103 | Not tested | Not tested | Not tested | 4.865 | Not tested | 1.543 | Not tested | Not tested | 5.472 |
| Orisakwe et al | 2016 | Nigeria | MyMy Herbal Flouride Teeth | 0.978 | 8.718 | Not tested | Not tested | Not tested | 7.997 | Not tested | 1.231 | Not tested | Not tested | 13.554 |
| Orisakwe et al | 2016 | Nigeria | MyMy Power Gel (Dental Fresh) | 0 | 12.672 | Not tested | Not tested | Not tested | 4.197 | Not tested | 0.001 | Not tested | Not tested | 9.692 |
| Orisakwe et al | 2016 | Nigeria | Nanny Herbal | 0.001 | 9.321 | Not tested | Not tested | Not tested | 4.672 | Not tested | 0 | Not tested | Not tested | 11.169 |
| Orisakwe et al | 2016 | Nigeria | Oral B Pro-Expert Dent Fresh | 0.144 | 4.514 | Not tested | Not tested | Not tested | 2.283 | Not tested | 0.596 | Not tested | Not tested | 7.979 |
| Orisakwe et al | 2016 | Nigeria | Pepsodent | 0.284 | 23.575 | Not tested | Not tested | Not tested | 12.712 | Not tested | 0 | Not tested | Not tested | 18.631 |
| Orisakwe et al | 2016 | Nigeria | Red Olive Gel | 1.284 | 15.369 | Not tested | Not tested | Not tested | 10.891 | Not tested | 5.968 | Not tested | Not tested | 8.421 |
| Orisakwe et al | 2016 | Nigeria | Sensodyne Gentle Whitening | 0.484 | 10.794 | Not tested | Not tested | Not tested | 3.101 | Not tested | 0.031 | Not tested | Not tested | 12.235 |
| Orisakwe et al | 2016 | Nigeria | Sensodyne Original St. Chloride | 0.456 | 9.973 | Not tested | Not tested | Not tested | 3.412 | Not tested | 1.385 | Not tested | Not tested | 10.897 |
| Orisakwe et al | 2016 | Nigeria | Sensodyne (Tooth Care F) | 0.453 | 10.321 | Not tested | Not tested | Not tested | 2.978 | Not tested | 0 | Not tested | Not tested | 11.872 |
| Orisakwe et al | 2016 | Nigeria | Aqua Fresh Mink Mint | 0.342 | 13.694 | Not tested | Not tested | Not tested | 5.172 | Not tested | 0.032 | Not tested | Not tested | 14.179 |
| Orisakwe et al | 2016 | Nigeria | Aqua Fresh Minty | 2.238 | 15.65 | Not tested | Not tested | Not tested | 13.157 | Not tested | 10.706 | Not tested | Not tested | 18.219 |
| Orisakwe et al | 2016 | Nigeria | Close Up Complete | 0 | 6.329 | Not tested | Not tested | Not tested | 2.698 | Not tested | 4.116 | Not tested | Not tested | 11.821 |
| Orisakwe et al | 2016 | Nigeria | Close Up Fine Breeze | 0 | 6.365 | Not tested | Not tested | Not tested | 1.055 | Not tested | 0 | Not tested | Not tested | 10.655 |
| Orisakwe et al | 2016 | Nigeria | Colgate Fresh Confidence (External Red) | 0.249 | 12.234 | Not tested | Not tested | Not tested | 3.324 | Not tested | 0.024 | Not tested | Not tested | 10.52 |
| Orisakwe et al | 2016 | Nigeria | Colgate Fresh Confidence (Main Gate) | 0.543 | 11.439 | Not tested | Not tested | Not tested | 4.123 | Not tested | 0.012 | Not tested | Not tested | 11.253 |
| Orisakwe et al | 2016 | Nigeria | Colgate (Great Regular Flavour) | 1.316 | 13.482 | Not tested | Not tested | Not tested | 3.431 | Not tested | 0 | Not tested | Not tested | 8.975 |
| Orisakwe et al | 2016 | Nigeria | Colgate Herbal | 2.218 | 16.314 | Not tested | Not tested | Not tested | 11.554 | Not tested | 3.217 | Not tested | Not tested | 18.535 |
| Orisakwe et al | 2016 | Nigeria | Colgate Junior | 0.363 | 13.13 | Not tested | Not tested | Not tested | 2.742 | Not tested | 0 | Not tested | Not tested | 9.992 |
| Orisakwe et al | 2016 | Nigeria | Colgate Strengthen, Teeth Freshen | 0.331 | 10.842 | Not tested | Not tested | Not tested | 2.114 | Not tested | 0.01 | Not tested | Not tested | 13.863 |
| Orisakwe et al | 2016 | Nigeria | Colgate Total | 0.292 | 9.401 | Not tested | Not tested | Not tested | 3.102 | Not tested | 0 | Not tested | Not tested | 9.013 |
| Orisakwe et al | 2016 | Nigeria | Colgate Triple Action | 0.214 | 11.369 | Not tested | Not tested | Not tested | 2.341 | Not tested | 0.019 | Not tested | Not tested | 12.023 |
| Orisakwe et al | 2016 | Nigeria | Flodent | 2.49 | 18.092 | Not tested | Not tested | Not tested | 16.336 | Not tested | 10.854 | Not tested | Not tested | 18.146 |
| Orisakwe et al | 2016 | Nigeria | Meriadent P Protection | 0.034 | 14.443 | Not tested | Not tested | Not tested | 4.476 | Not tested | 0.022 | Not tested | Not tested | 11.986 |
| Orisakwe et al | 2016 | Nigeria | Oral B | 0.412 | 10.438 | Not tested | Not tested | Not tested | 4.031 | Not tested | 5.324 | Not tested | Not tested | 13.142 |
| Salama AK* | 2016 | Saudi Arabia | Oral B | 10.34±0.2 | 5069.22±25.8 | 9.28±0.009 | 0.6±0.0006 | Not tested | 47.52±3.3 | Not tested | 1434.7±17.2 | 12745.92±280.4 | Not tested | 212.22±1.3 |
| Salama Ak* | 2016 | Saudi Arabia | Crest | 18.28±0.3 | 4037±80.7 | 13.14±0.1 | 12.74±0.2 | Not tested | 57.8±3.8 | Not tested | 4762.8±23.8 | 22988.52±459.8 | Not tested | 222.02±4.7 |
| Salama AK* | 2016 | Saudi Arabia | Colgate | 55.28±0.6 | 6313±63.1 | 0 | 26.94±0.5 | Not tested | 2608.88±26.1 | Not tested | 1041.98±9.4 | 5590.92±72.7 | Not tested | 1557.24±12.5 |
| Salama AK* | 2016 | Saudi Arabia | Dabur Herbi | 2.08±0.008 | 1856.34±14.9 | 3.34±0.02 | 2.4±0.005 | Not tested | 17.66±0.3 | Not tested | 838.80±1.7 | 6473.02±58.3 | Not tested | 73.60±1.3 |
| Vella & Attard | 2019 | Malta | Conventional 1 | 0 | 3.26±0.152 | 0 | Not tested | 5.29±0.018 | Not tested | 1842±3.55 | 1.42±0.012 | 1.35±0.006 | 1.76±0.012 | 1.96±0.007 |
| Vella & Attard | 2019 | Malta | Conventional 2 | 0 | 8.83±0.019 | 0 | Not tested | 3.36±0.017 | Not tested | 2417±0.859 | 7.35±0.019 | 3.68±0.003 | 17.68±0.0088 | 1.31±0.006 |
| Vella & Attard | 2019 | Malta | Conventional 3 | 0 | 2.37±0.064 | 0 | Not tested | 3.26±0.035 | Not tested | 7.8±1.69 | 1.35±0.026 | 0.84±0.003 | 12.33 | 1.34±0.02 |
| Vella & Attard | 2019 | Malta | Herbal 4 | 0 | 12.04±0.079 | 0 | Not tested | 5.12±0.02 | Not tested | 2.9±0.072 | 0.72±0.01 | 1.4±0.003 | 4.5±0.058 | 0.43±0.0033 |
| Vella & Attard | 2019 | Malta | Herbal 5 | 0 | 2.23±0.05 | 0 | Not tested | 2.23±0.089 | Not tested | 3.66±0.051 | 0.67±0.012 | 1.26±0.006 | 7.84±0.017 | 1.55±0.021 |
| Vella & Attard | 2019 | Malta | Herbal 6 | 0 | 4.33±0.133 | 0 | Not tested | 3.1±0.081 | Not tested | 6.32±0.076 | 0.65±0.015 | 1.16 | 9.68±0.009 | 1.99±0.032 |
| Vella & Attard | 2019 | Malta | Children 7 | 0 | 2.64±0.117 | 0 | Not tested | 2±0.015 | Not tested | 0.31±0.01 | 0.4±0.023 | 0.81 | 8.52±0.017 | 1.73±0.013 |
| Vella & Attard | 2019 | Malta | Children 8 | 0 | 4.76±0.062 | 0 | Not tested | 3.44±0.029 | Not tested | 3.12±0.074 | 0.28±0.01 | 0.73±0.003 | 10.2±0.009 | 1.15±0.009 |
| Vella & Attard | 2019 | Malta | Children 9 | 0 | 4.72±0.065 | 0 | Not tested | 3.82±0.052 | Not tested | 0 | 0.86±0.009 | 1.37±0.003 | 7.56±0.007 | 2.54±0.035 |
| Arshad et al | 2020 | Pakistan | TP 1 | 0.3039 | Not tested | Not tested | Not tested | Not tested | Not tested | Not tested | Not tested | Not tested | Not tested | Not tested |
| Arshad et al | 2020 | Pakistan | TP 2 | 0.0037 | Not tested | Not tested | Not tested | Not tested | Not tested | Not tested | Not tested | Not tested | Not tested | Not tested |
| Arshad et al | 2020 | Pakistan | TP 3 | 0.047 | Not tested | Not tested | Not tested | Not tested | Not tested | Not tested | Not tested | Not tested | Not tested | Not tested |
| Arshad et al | 2020 | Pakistan | TP 4 | 0.0302 | Not tested | Not tested | Not tested | Not tested | Not tested | Not tested | Not tested | Not tested | Not tested | Not tested |
| Arshad et al | 2020 | Pakistan | TP 5 | 0.037 | Not tested | Not tested | Not tested | Not tested | Not tested | Not tested | Not tested | Not tested | Not tested | Not tested |
| Arshad et al | 2020 | Pakistan | TP 6 | 0 | Not tested | Not tested | Not tested | Not tested | Not tested | Not tested | Not tested | Not tested | Not tested | Not tested |
| Arshad et al | 2020 | Pakistan | TP 7 | 0 | Not tested | Not tested | Not tested | Not tested | Not tested | Not tested | Not tested | Not tested | Not tested | Not tested |
| Arshad et al | 2020 | Pakistan | TP 8 | 0.0125 | Not tested | Not tested | Not tested | Not tested | Not tested | Not tested | Not tested | Not tested | Not tested | Not tested |
| Arshad et al | 2020 | Pakistan | TP 9 | 0 | Not tested | Not tested | Not tested | Not tested | Not tested | Not tested | Not tested | Not tested | Not tested | Not tested |
| Arshad et al | 2020 | Pakistan | TP 10 | 0 | Not tested | Not tested | Not tested | Not tested | Not tested | Not tested | Not tested | Not tested | Not tested | Not tested |
| Arshad et al | 2020 | Pakistan | TP 11 | 0 | Not tested | Not tested | Not tested | Not tested | Not tested | Not tested | Not tested | Not tested | Not tested | Not tested |
| Arshad et al | 2020 | Pakistan | TP 12 | 0 | Not tested | Not tested | Not tested | Not tested | Not tested | Not tested | Not tested | Not tested | Not tested | Not tested |
| Arshad et al | 2020 | Pakistan | TP 13 | 0 | Not tested | Not tested | Not tested | Not tested | Not tested | Not tested | Not tested | Not tested | Not tested | Not tested |
| Arshad et al | 2020 | Pakistan | TP 14 | 1.345 | Not tested | Not tested | Not tested | Not tested | Not tested | Not tested | Not tested | Not tested | Not tested | Not tested |
| Arshad et al | 2020 | Pakistan | TP 15 | 1.296 | Not tested | Not tested | Not tested | Not tested | Not tested | Not tested | Not tested | Not tested | Not tested | Not tested |
| Arshad et al | 2020 | Pakistan | TP 16 | 0.3367 | Not tested | Not tested | Not tested | Not tested | Not tested | Not tested | Not tested | Not tested | Not tested | Not tested |
| Arshad et al | 2020 | Pakistan | TP 17 | 0.647 | Not tested | Not tested | Not tested | Not tested | Not tested | Not tested | Not tested | Not tested | Not tested | Not tested |
| Arshad et al | 2020 | Pakistan | TP 18 | 1.392 | Not tested | Not tested | Not tested | Not tested | Not tested | Not tested | Not tested | Not tested | Not tested | Not tested |
| Paul et al | 2020 | Bangladesh | Pepsodent | Not tested | 0.88±0.09 | Not tested | 0.044±0.02 | Not tested | Not tested | Not tested | Not tested | 4.64±0.78 | Not tested | Not tested |
| Paul et al | 2020 | Bangladesh | Close up | Not tested | 0.77±0.1 | Not tested | 0.085±0.03 | Not tested | Not tested | Not tested | Not tested | 4.26±0.59 | Not tested | Not tested |
| Paul et al | 2020 | Bangladesh | White plus | Not tested | 0.27±0.03 | Not tested | 0.027±0.01 | Not tested | Not tested | Not tested | Not tested | 2.78±0.48 | Not tested | Not tested |
| Paul et al | 2020 | Bangladesh | Fresh gel | Not tested | 1.34±0.12 | Not tested | 0.224±0.08 | Not tested | Not tested | Not tested | Not tested | 5.63±1.02 | Not tested | Not tested |
| Paul et al | 2020 | Bangladesh | Colgate active salt | Not tested | 1.27±0.15 | Not tested | 0.153±0.05 | Not tested | Not tested | Not tested | Not tested | 5.38±0.96 | Not tested | Not tested |
| Paul et al | 2020 | Bangladesh | Medi plus | Not tested | 2.12±0.26 | Not tested | 0.637±0.12 | Not tested | Not tested | Not tested | Not tested | 13.1±1.68 | Not tested | Not tested |
| Paul et al | 2020 | Bangladesh | Pepsodent herbal | Not tested | 1.23±0.22 | Not tested | 0.098±0.04 | Not tested | Not tested | Not tested | Not tested | 5.02±1.12 | Not tested | Not tested |
| Paul et al | 2020 | Bangladesh | Colgate herbal | Not tested | 0.34±0.01 | Not tested | 0.183±0.07 | Not tested | Not tested | Not tested | Not tested | 4.49±0.51 | Not tested | Not tested |
| Paul et al | 2020 | Bangladesh | Meril baby | Not tested | 0.98±0.1 | Not tested | 0.042±0.01 | Not tested | Not tested | Not tested | Not tested | 4.37±0.39 | Not tested | Not tested |
| Paul et al | 2020 | Bangladesh | Kodomo | Not tested | 0.57±0.06 | Not tested | 0.076±0.03 | Not tested | Not tested | Not tested | Not tested | 3.71±0.88 | Not tested | Not tested |
| Ogidi & Agbo | 2021 | Nigeria | TP 1 | Not tested | Not tested | Not tested | Not tested | Not tested | Not tested | 84.67±1.83 | Not tested | Not tested | Not tested | Not tested |
| Ogidi & Agbo | 2021 | Nigeria | TP 2 | Not tested | Not tested | Not tested | Not tested | Not tested | Not tested | 81.27±0.86 | Not tested | Not tested | Not tested | Not tested |
| Ogidi & Agbo | 2021 | Nigeria | TP 3 | Not tested | Not tested | Not tested | Not tested | Not tested | Not tested | 1.19±0.06 | Not tested | Not tested | Not tested | Not tested |
| Ogidi & Agbo | 2021 | Nigeria | TP 4 | Not tested | Not tested | Not tested | Not tested | Not tested | Not tested | 1.88±0.03 | Not tested | Not tested | Not tested | Not tested |
| Ogidi & Agbo | 2021 | Nigeria | TP 5 | Not tested | Not tested | Not tested | Not tested | Not tested | Not tested | 3.08±0.02 | Not tested | Not tested | Not tested | Not tested |
| Almukainzi et al | 2022 | Saudi Arabia | TP 1 | 9.19±13 | 75.86±107.27 | Not tested | 221.96±313.9 | Not tested | 17.65±24.95 | 556.67±787.23 | 1610.64±2277.26 | 264±373.3 | 16826±23791.75 | 970.83±1372.89 |
| Almukainzi et al | 2022 | Saudi Arabia | TP 2 | 8.8±12.44 | 78.31±110.75 | Not tested | 209.33±296.04 | Not tested | 18.29±25.86 | 1114.37±1575.89 | 878.37±1242.06 | 269.94±381.66 | 15603.46±22066.21 | 600.37±849.01 |
| Valentine & Ozioma | 2022 | Nigeria | TP 1 gel | 0 | 0 | Not tested | Not tested | Not tested | Not tested | 3.92±0.89 | 0.05±0.04 | 1.47±0.03 | 2.06±0.22 | 0.08±0.02 |
| Valentine & Ozioma | 2022 | Nigeria | TP 1 tube | 0 | 0 | Not tested | Not tested | Not tested | Not tested | 0.01±0.02 | 1.89±0.5 | 0 | 2.24±0.13 | 0 |
| Valentine & Ozioma | 2022 | Nigeria | TP 2 gel | 0.13±0.01 | 0.01±0.01 | Not tested | Not tested | Not tested | Not tested | 1.45±0.08 | 0 | 1.83±0.04 | 7.06±0.12 | 0.09±0.08 |
| Valentine & Ozioma | 2022 | Nigeria | TP 2 tube | 0 | 0 | Not tested | Not tested | Not tested | Not tested | 0.01±0.01 | 0.28±0.02 | 0 | 1.21±0.69 | 0.02±0.05 |
| Valentine & Ozioma | 2022 | Nigeria | TP 3 gel | 0 | 0.02 | Not tested | Not tested | Not tested | Not tested | 0.16±0.06 | 0.03±0.02 | 1.49±0.06 | 1.45±0.08 | 0.04±0.01 |
| Valentine & Ozioma | 2022 | Nigeria | TP 3 tube | 0 | 0 | Not tested | Not tested | Not tested | Not tested | 0 | 0 | 0 | 2.69±0.19 | 0.03±0.01 |
| Valentine & Ozioma | 2022 | Nigeria | TP 4 gel | 0 | 0 | Not tested | Not tested | Not tested | Not tested | 0.15±0.15 | 0 | 1.64±0.15 | 3.18±0.2 | 0 |
| Valentine & Ozioma | 2022 | Nigeria | TP 4 tube | 0.01±0 | 0 | Not tested | Not tested | Not tested | Not tested | 0.01±0.01 | 1.26±0.64 | 0 | 0 | 0 |
| Valentine & Ozioma | 2022 | Nigeria | TP 5 gel | 0 | 0 | Not tested | Not tested | Not tested | Not tested | 0.46±0.19 | 0 | 1.56±0.08 | 2.04±0.14 | 0.07±0.02 |
| Valentine & Ozioma | 2022 | Nigeria | TP 5 tube | 0 | 0 | Not tested | Not tested | Not tested | Not tested | 0 | 1.28±0.12 | 0 | 0 | 0 |
| Lawi et al | 2023 | Iraq | Himalaya mint fresh | Not tested | 9.04 | Not tested | Not tested | Not tested | Not tested | 11.3 | Not tested | Not tested | 72.5 | Not tested |
| Lawi et al | 2023 | Iraq | Dabur Neem | Not tested | 11.05 | Not tested | Not tested | Not tested | Not tested | 5.63 | Not tested | Not tested | 189.8 | Not tested |
| Lawi et al | 2023 | Iraq | Dabur Basil | Not tested | 4.02 | Not tested | Not tested | Not tested | Not tested | 3.18 | Not tested | Not tested | 147.94 | Not tested |
| Lawi et al | 2023 | Iraq | Dabur Olive | Not tested | 12.05 | Not tested | Not tested | Not tested | Not tested | 2.72 | Not tested | Not tested | 151.68 | Not tested |
| Lawi et al | 2023 | Iraq | Crest Chamomile | Not tested | 5.02 | Not tested | Not tested | Not tested | Not tested | 402.34 | Not tested | Not tested | 528.31 | Not tested |
| Lawi et al | 2023 | Iraq | Colgate Seaweed | Not tested | 1 | Not tested | Not tested | Not tested | Not tested | 1.59 | Not tested | Not tested | 63.67 | Not tested |
| Lawi et al | 2023 | Iraq | Colgate Fleur delotedeasia | Not tested | 5.02 | Not tested | Not tested | Not tested | Not tested | 6.1 | Not tested | Not tested | 57.82 | Not tested |
| Lawi et al | 2023 | Iraq | Dabur Blackseed | Not tested | 2.01 | Not tested | Not tested | Not tested | Not tested | 5.9 | Not tested | Not tested | 654.24 | Not tested |
| Lawi et al | 2023 | Iraq | Dabur Salt and lemon | Not tested | 10.04 | Not tested | Not tested | Not tested | Not tested | 3.05 | Not tested | Not tested | 155.43 | Not tested |
| Lawi et al | 2023 | Iraq | Dabur Aloe Vera | Not tested | 5.02 | Not tested | Not tested | Not tested | Not tested | 3.78 | Not tested | Not tested | 36.75 | Not tested |
| * values in ppb; all other values in ppm | | | | | | | | | | | | | | |
